# A Tragic Loss

**DOI:** 10.3402/ijch.v73.25267

**Published:** 2014-07-04

**Authors:** Kue Young

The circumpolar health community lost a much respected and loved friend, colleague and mentor on 17 June, 2014. Dr. Éric Dewailly, aged 59, a professor at Laval University in Québec, Canada, died in a tragic rockslide on the island of Réunion in the Indian Ocean, while holidaying with his family.

Eric was well known to many researchers across the Arctic. He was a superb pioneering scientist who made significant contributions to environmental health science and the epidemiology of chronic diseases. He worked among the Inuit in the Nunavik region of northern Québec for decades. The people there fondly joked about his strong French accent. His research not only contributed to the body of scientific knowledge but also had an impact on public health policy and practice. Throughout the years, he mentored scores of graduate students, making his research centre in Québec a most active incubator of research talent.

Born in France, Eric studied at the University of Lille, where he graduated with a doctorate in medicine in 1982. He came to Canada in 1983 and studied at Laval University where he completed a residency in community medicine and obtained an MSc in epidemiology in 1987. He returned to Lille for his PhD in toxicology (1990). In 1998, he headed the Public Health Research Unit at the *Centre hospitalier universitaire de Québec*, from where he conducted major studies in northern Québec. He was principal investigator of *Nasivvik – Centre for Inuit Health and Changing Environments* funded by the Canadian Institutes of Health Research. He also had broad research interests in global health and had conducted studies in the Caribbean, French Polynesia and sub-Saharan Africa.

In a news release, Minnie Grey, the executive director of the Nunavik Regional Board of Health and Social Services, wrote: “Eric was a generous, kind and inspirational man, always available for the Inuit, and his tremendous work helped us in a way that words cannot fully express. His unique knowledge, his love for the North and his presence will be greatly missed by all.”

Eric loved the seas, and sailing was his passion. That he died in a beautiful tropical island offers some comfort to us.

*Kue Young* Editor-in-Chief

